# Transcriptomic Evidence That Switching from Tobacco to Electronic Cigarettes Does Not Reverse Damage to the Respiratory Epithelium

**DOI:** 10.3390/toxics10070370

**Published:** 2022-07-04

**Authors:** Giovanna L. Pozuelos, Meenakshi Kagda, Matine A. Rubin, Maciej L. Goniewicz, Thomas Girke, Prue Talbot

**Affiliations:** 1 Department of Molecular, Cell and Systems Biology, University of California, Riverside, CA 92521, USA; gpozu001@ucr.edu (G.L.P.); mkagd001@ucr.edu (M.K.); mrubi017@ucr.edu (M.A.R.); 2 Department of Health Behavior, Roswell Park Comprehensive Cancer Center, Buffalo, NY 14203, USA; maciej.goniewicz@roswellpark.org; 3 Institute for Integrative Genome Biology, University of California, Riverside, CA 92521, USA; thomas.girke@ucr.edu

**Keywords:** electronic cigarettes, cigarette smoke, transcriptomic analysis, immune response, oxidative stress, squamous metaplasia, ciliogenesis

## Abstract

The health benefits of switching from tobacco to electronic cigarettes (ECs) are neither confirmed nor well characterized. To address this problem, we used RNA-seq analysis to compare the nasal epithelium transcriptome from the following groups (*n* = 3 for each group): (1) former smokers who completely switched to second generation ECs for at least 6 months, (2) current tobacco cigarette smokers (CS), and (3) non-smokers (NS). Group three included one former cigarette smoker. The nasal epithelial biopsies from the EC users vs. NS had a higher number of differentially expressed genes (DEGs) than biopsies from the CS vs. NS and CS vs. EC sets (1817 DEGs total for the EC vs. NS, 407 DEGs for the CS vs. NS, and 116 DEGs for the CS vs. EC comparison). In the EC vs. NS comparison, enriched gene ontology terms for the downregulated DEGs included cilium assembly and organization, whereas gene ontologies for upregulated DEGs included immune response, keratinization, and NADPH oxidase. Similarly, ontologies for cilium movement were enriched in the downregulated DEGs for the CS vs. NS group. Reactome pathway analysis gave similar results and also identified keratinization and cornified envelope in the upregulated DEGs in the EC vs. NS comparison. In the CS vs. NS comparison, the enriched Reactome pathways for upregulated DEGs included biological oxidations and several metabolic processes. Regulator effects identified for the EC vs. NS comparison were inflammatory response, cell movement of phagocytes and degranulation of phagocytes. Disease Ontology Sematic Enrichment analysis identified lung disease, mouth disease, periodontal disease and pulmonary fibrosis in the EC vs. NS comparison. Squamous metaplasia associated markers, keratin 10, keratin 13 and involucrin, were increased in the EC vs. NS comparison. Our transcriptomic analysis showed that gene expression profiles associated with EC use are not equivalent to those from non-smokers. EC use may interfere with airway epithelium recovery by promoting increased oxidative stress, inhibition of ciliogenesis, and maintaining an inflammatory response. These transcriptomic alterations may contribute to the progression of diseases with chronic EC use.

## 1. Introduction

Electronic cigarettes (ECs) are popular battery-operated devices, which aerosolize e-liquids (nicotine or non-nicotine containing flavored mixtures) using a heating coil [1,2]. Some studies have suggested that EC use may be a healthy long-term alternative to cigarette smoking that can help reverse and prevent progression of tobacco related respiratory diseases [3,4,5,6,7,8]. Some cigarette smokers who became EC users or dual users have a self-reported reduction in respiratory symptoms after switching [3,5,6,9]. In contrast, others have self-reported that respiratory symptoms worsen with EC use [10,11]. In addition, a number of case studies have linked EC use to adverse effects in the respiratory, gastrointestinal, cardiovascular, neurological and immune systems [12]. In 2019, an outbreak of e-cigarette or vaping-related acute lung injury (EVALI) was reported in the United States. Patients with EVALI reported adverse respiratory symptoms such as coughing, dyspnea, pleuritic chest pain as well as abnormal pulmonary radiographic phenotypes [13,14,15]. Lung injuries included acute eosinophilic pneumonia and diffuse alveolar damage [16].

To better understand the effect of vaping nicotine on pulmonary health, transcriptomic studies have examined gene expression in EC users. These studies differ in the techniques used in comparison groups, and the generation of ECs used is sometimes variable or not given. A prior study used a Nanostring immune expression panel to analyze nasal epithelial biopsies from non-smokers, cigarette smokers and former cigarette users who had switched to ECs for at least 6 months (this group included dual users). This study identified decreased expression of immune-related genes in the EC users and cigarette users compared to the non-smokers [17]. In another study, immune responses were altered in the EC group, which included former smokers and dual users, following inoculation with live-attenuated influenza virus [18].

Bronchial airway epithelial brushings were analyzed from former smokers, tobacco cigarette smokers and former smokers who switched to ECs for at least 3 months using whole-transcriptomic arrays [19]. This study did not contain non-smokers. Here, interleukin receptor complex associated genes were upregulated, whereas genes involved in cilia were downregulated in EC users compared to former smokers [19]. Variations in the results of these EC studies may be due to differences in the compositions of the groups, puffs/day, cigarette/day, the generation of ECs used, flavor chemicals, and overall differences in experimental designs.

The purpose of this study was to determine if switching from combustible cigarettes to ECs restored nasal epithelium to that of a non-smoker. This was accomplished by comparing gene expression in nasal epithelial biopsies from individuals who were: (1) non-smokers (NS group), (2) exclusive tobacco cigarette smokers (CS group), and (3) exclusive EC users who had switched completely from tobacco cigarettes to second generation nicotine-containing ECs for at least 6 months (EC group). Our transcriptomic analysis showed that some gene expression profiles in the EC group had not reverted to those found in the non-smoker group and suggest that long-term use of ECs may contribute to the progression of several respiratory diseases.

## 2. Materials and Methods

### 2.1. Sample Collection and RNA Extraction

Nasal biopsies from 22 human participants were collected in RNA Later (Qiagen, Hilden, Germany 76104) and frozen at Roswell Park Cancer Institute, Buffalo, New York. These were shipped frozen to the University of California, Riverside and stored at −80 °C until analyzed. For RNA extraction, individual samples were thawed on ice prior to further processing. The samples were washed twice using Dulbecco’s phosphate-buffered saline (Lonza, Basel, Switzerland Cat. No. 17-513F). Qiagen RNeasy Plus Microkit (Cat No./ID: 74304) was used to extract total RNA, which was resuspended in 15 µL of RNase free distilled water. Sterile Eppendorf tubes containing RNA samples were snap-frozen in liquid nitrogen before storing at −80 °C.

### 2.2. Sample Information, Selection, and Demographics

Information regarding smoking history was obtained from 19 female participants recruited into the study, and the concentrations of urine cotinine were measured at Roswell Park (Table S1). Because gene expression can differ with gender [20], we focused on one gender (females) to maximize the possibility of finding significant differences in gene expression given our small sample size. The cotinine concentrations were used to exclude NS participants that tested positive and to include those in the CS and EC group with medium to high concentrations of cotinine. From the remaining participants, three participants were selected from each of the groups (NS, EC, and CS) for transcriptomic analysis. Selection of participants was based on matching age and ethnicity. Those included for transcriptomic analysis were white females below the age of 50 that had urine cotinine levels of at least 400 ng/mL (CS (*n* = 3) and EC (*n* = 3) groups) and undetectable cotinine (<0.25 ng/mL; NS (*n* = 3) group).

### 2.3. Library Preparation and Sequencing

RNA from all samples was extracted in house and shipped to Cofactor Genomics (St. Louis, MO, USA) on dry ice for library preparation and sequencing. RNA quality was checked using a Bioanalyzer. Samples having good RIN numbers (>7) were selected for RNA-seq library preparation (mRNAble Cat No. CFG001-30) and subsequent sequencing steps were performed by Cofactor Genomics. Total RNA reverse transcription was performed with an Oligo(Dt) primer followed by limited cDNA amplification using the SMARTer^®^ Ultra^®^ Low Input RNA Kit for Sequencing–v4 (Takara Bio USA, Inc., Mountain View, CA, USA). Next, the full-length cDNA was subject to fragmentation followed by tagging. The final sequencing cDNA library was generated using limited PCR enrichment (Nextera^®^ XT DNA Library Prep, Illumina, San Diego, CA, USA). Single-end 75 base pair reads were generated using the Illumina NextSeq500 platform. Resulting FASTQ files containing all the reads were provided to us on a URL.

### 2.4. Hierarchical Clustering and Differential Expression Analysis

The FASTQ files were processed at the University of California on a High-Performance Computing Cluster (HPCC). The files were preprocessed to remove adapter sequences and low-quality score reads using the Trimmomatic package [21]. The alignment, raw read count steps, and differential gene calling steps were performed using a pre-built RNA-seq workflow known as systemPipeR [22]. The Human reference genome (UCSC hg19) was obtained from Illumina iGenomes (ftp.illumina.com (accessed on 10 October 2019)). Preprocessed reads were then aligned to the reference genome using Tophat2 (2.0.14) [23,24]. Raw read counts for exonic gene regions as well as normalized RPKM (reads per kilobase per million) values were calculated with the summarized Overlaps function of the GenomicAlignment package from Bioconductor. Genes having low expression counts were removed using a cut-off of an average of one CPM across all samples leaving a total of 15,440 genes for analysis. Hierarchical clustering was performed with hclust function using Spearman’s correlation values as a similarity metric to identify outliers within each of the groups. After outliers were removed, each group (EC, NS and CS) had three individuals. Analysis of differentially expressed genes (DEGs) was performed with edgeR comparing EC vs. NS, CS vs. NS and CS vs. EC [25]. Raw FASTQ files were deposited under accession PRJNA666452in the Sequence Read Archive (SRA) of the National Center for Biotechnology Information. DEGs for all comparisons are in supplementary tables (Tables S2–S4).

### 2.5. Gene Ontology (GO) Enrichment and Reactome Pathway Analyses

Enrichment analysis was performed for GO terms (clusterProfiler package from R/Bioconductor) [25,26], pathways (ReactomePA package) [27,28], and disease (DOSE—Disease Ontology Sematic and Enrichment) [29]. In all cases, over-representation analysis with the Fisher’s exact test was used as a statistical test. The required gene sets were obtained from the DEG analysis results by applying the following filter setting: logFC ≥ 1 (upregulated; or ≤−1 for downregulated) and an FDR ≤ 0.05. Over-represented GO terms, pathways, and disease terms had to have an adjusted *p*-value ≤ 0.05 using the Bonferroni Hochberg method for multiple testing correction. The heatmap was generated with the pheatmap package using z-scaled RPKM values.

### 2.6. Ingenuity Pathway Analysis (IPA)

IPA software (QIAGEN Inc., Germantown, MD, USA, https://digitalinsights.qiagen.com/IPA (accessed on 1 September 2021)) was used to identify enriched Diseases and Biological Functions and Regulator Effects. A core analysis was performed using the “human” and “lung system” prefilters. Cutoff values were set as follows: log-fold change +/−0.6, *p*-value 0.05, and q-value 0.05. Significance of Disease and Biological Functions was determined using a *p*-value calculated with a right-tailed Fisher’s exact test. Molecular networks were generated using the “Path Explorer” function, which identified molecular targets in our data associated with “e-cigarette aerosol”, “nicotine” or “cigarette smoke” exposure and their downstream effect on biological functions. The “Molecule Activity Predictor” tool predicted the activation state of biological functions. Regulator Effects were extracted to identify upstream regulators and biological processes affected in our data set.

## 3. Results

### 3.1. Differential Gene Expression (DEGs) in EC, CS, and NS: Initial Analysis

**Figure 1:**
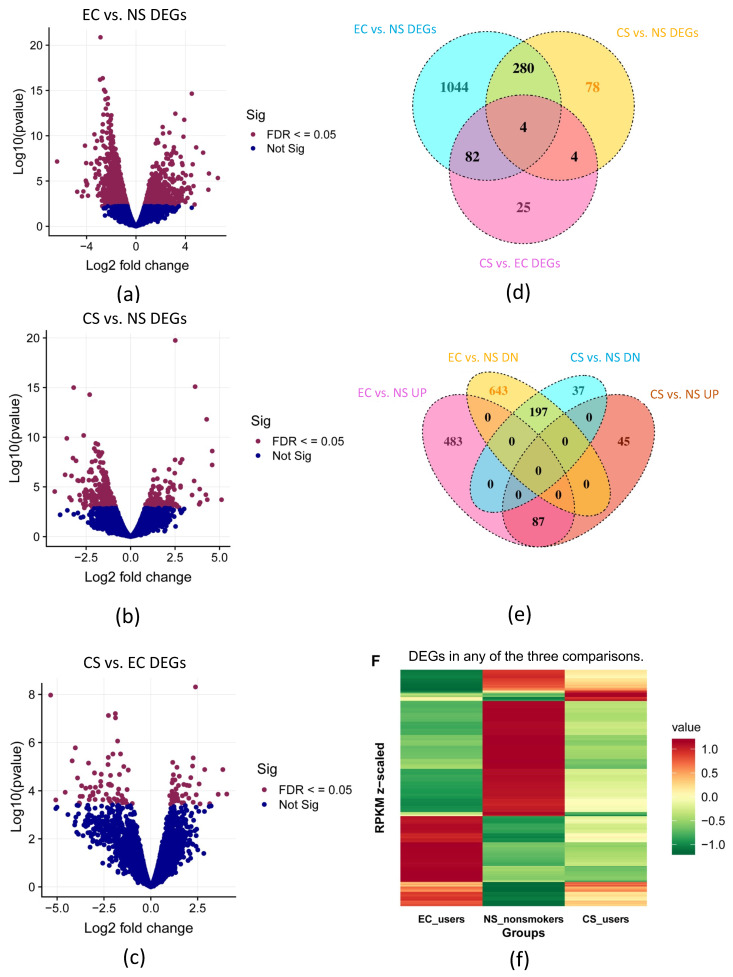
Differential gene expression in the EC, NS, and CS groups. Volcano plots showing DEGs (red dots) in (**a**) EC vs. NS, (**b**) CS vs. NS, (**c**) CS vs. EC sets (FDR < 0.05). (**d**) Venn diagram showing all the DEGs in the EC, NS and CS groups. (**e**) Venn diagram comparing the up and downregulated DEGs (FDR < 0.05) in the EC vs. NS, CS vs. NS, CS vs. EC sets. (**f**) Heatmap showing the RPKM Z-scores for the DEGs that were significant (FDR < 0.05) in any of the three comparisons. Transcripts are different in the three groups, and the EC and NS groups have opposite responses (**f**).

For the initial analysis, we used DEG sets obtained with an FDR ≤ 0.05 and a log2 fold change (LFC) ≥ 1.0. DEGs are shown for each of the three comparisons (EC vs. NS, CS vs. NS, CS vs. EC) in the volcano plots (Figure 1a–c). The highest number of DEGs (1410 total, 570 upregulated and 1243 downregulated) was in the EC vs. NS comparison (Figure 1a). Within the CS vs. NS comparison, a total of 366 DEGs were found (132 upregulated and 281 downregulated) (Figure 1b). Fewer genes were differentially expressed in the CS vs. EC comparison (115 total, 52 up and 64 downregulated) (Figure 1c). Figure 1d shows the number of DEGs that were in common in the three comparisons. The greatest overlap in DEGs was in the EC vs. NS and CS vs. NS comparisons (Figure 1d). The number of DEGs that were up and downregulated in the EC vs. NS and CS vs. NS groups are shown in Figure 1e. The highest number of overlapping DEGs was in the downregulated sets (Figure 1e). The expression pattern of the RPKM z-scores of the individual DEGs was different in the three groups (Figure 1f).

### 3.2. GO Term Enrichment Analysis for EC vs. NS, CS vs. NS, and CS vs. EC

**Figure 2:**
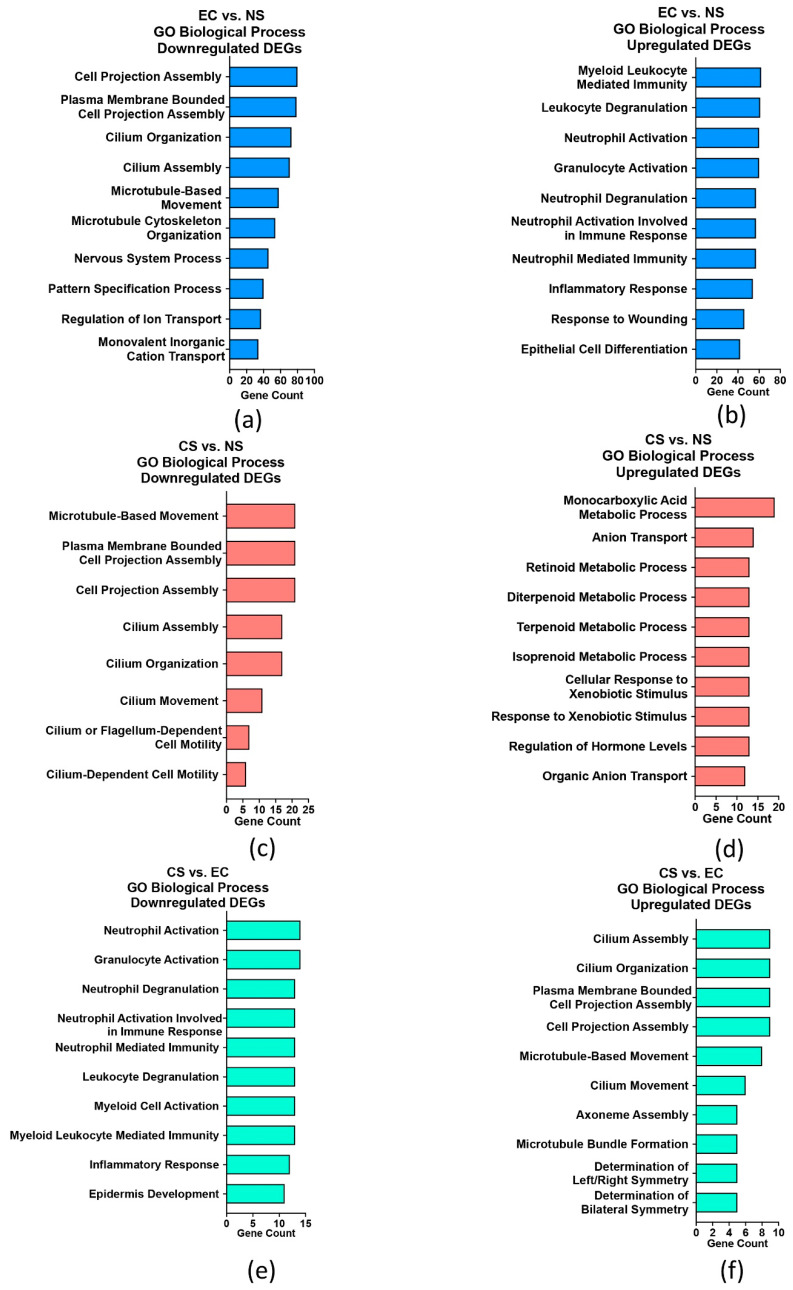
Top 10 enriched GO term annotations for each comparison. (**a**) Enriched downregulated biological processes in the EC vs. NS set. (**b**) Enriched upregulated biological processes in the EC vs. NS set. (**c**) Enriched downregulated biological processes in the CS vs. NS set. (**d**) Enriched upregulated biological processes in the CS vs. NS set. (**e**) Enriched downregulated biological processes in the CS vs. EC set. (**f**) Enriched upregulated biological processes in the CS vs. EC set. (FDR < 0.05). Biological processes were mainly related to cilia assembly (EC, CS) and immune response (EC).

GO terms over-represented in the DEGs were identified using ClusterProfiler [25] to obtain an overview of the processes affected in each group (Figure 2 and Tables S5–S7). Most of the enriched terms within the biological processes category for downregulated genes in the EC vs. NS comparison included cilium organization (GO:0044782), cilium assembly (GO:0060271), cilium movement (GO:0003341), microtubule-based movement (GO:0007018), and plasma membrane bounded cell projection assembly (GO:0120031) (Figure 2a and Table S5A). Other biological processes included: nervous system process (GO:0050877), regulation of smoothened signaling pathway (GO:0008589), regulation of membrane potential (GO:0042391), and regulation of ion transport (GO:0043269) (Figure 2a and Table S5A). For the downregulated DEGs, enriched cellular components included cilia-related terms and molecular functions included ion channel activity (GO:0005509, GO:0005216) and microtubule motor activity (GO:0003774, GO:0003777, GO:1990939, GO:0008569) (Table S5B,C).

Over-represented biological processes for upregulated genes in the EC vs. NS comparison included inflammatory responses, such as neutrophil activation (GO:0042119), granulocyte activation (GO:0036230), and chronic inflammatory response (GO:0002544) (Figure 2b and Table S5D). Other processes included response/regulation to wound healing (GO:0042060), response to bacterium (GO:0009611), blood coagulation and its regulation (GO:0030193), response to xenobiotic stimulus (GO:0071466), and keratinization (GO:0031424) (Table S5D). The cellular component and molecular function terms for the upregulated DEGs included NADPH oxidase complex (GO:0043020) and superoxide-generating NADPH oxidase activator activity (GO:0008106) (Table S5E,F).

In the CS vs. NS comparison, the biological processes and cellular components in the downregulated DEGs were similar to the EC vs. NS group and included cilium assembly and organization (GO:0060271: GO:0044782: GO:0097014; GO:0005929) (Table S6A,B). The molecular functions for the downregulated genes included ATP-dependent microtubule motor activity minus-end-directed (GO:0008569), dynein light chain binding (GO:0045503), dynein intermediate chain binding (GO:0045505), and ATP-dependent microtubule motor activity (GO:1990939) (Table S6C). The upregulated biological processes included metabolic processes, such as monocarboxylic acid metabolic process (GO:0032787), anion transport (GO:0006820), and cellular response to xenobiotic stimulus (GO:0071466) (Figure 2d, Table S6D). The enriched cellular components for the upregulated genes, were very-low-density lipoprotein particle (GO:0034361) and triglyceride-rich plasma lipoprotein particle (GO:0034385) (Table S6E). The molecular functions included glucuronosyltransferase activity (GO:0015020), monocarboxylic acid binding (GO:0033293), carboxylic acid binding (GO:0031406), organic acid binding (GO:0043177), and alcohol dehydrogenase (NADP+) activity (GO:0008106) (Table S6F).

The biological processes in the CS vs. EC comparison for the downregulated genes included terms related to immune response, such as neutrophil activation (GO:0042119) and granulocyte activation (GO:0036230) (Figure 2e and Table S7A). The cellular components for downregulated DEGs included secretory granule membrane (GO:0030667), keratin filament (GO:0045095) and intermediate filament (GO:0005882) (Table S7B). The biological processes and cellular components for upregulated genes were related to cilia assembly and movement (GO:0003341; GO:0060271; GO:0044782; GO:0097014, GO:0005929, GO:0044441) (Figure 2f, Table S7D,E). Dynein light chain binding (GO:0045503) was enriched by the upregulated DEGs (Table S7F).

### 3.3. IPA Biological Functions for EC vs. NS

Biological functions analysis of lung-filtered data was conducted using IPA to make the analysis more specific to the lung and to obtain directionality. There was good agreement with results from the GO term analysis (Table S5). The biological functions analysis in IPA for the EC vs. NS comparison showed an increased predicted activation state (z-score > 2) for processes related to inflammatory response (e.g., response of myeloid cells, degranulation of phagocytes) and a decreased activation state (z-score < 2) for cilia formation. Other categories with increased activation states included cell-to-cell signaling, cell movement, and lipid metabolism (Table S8). The other comparisons (CN vs. NS and CN vs. EC) did not have biological functions with a significant z-score (>2.0).

### 3.4. Reactome Pathway Enrichment Analysis for EC vs. NS, CS vs. NS and CS vs. EC

**Figure 3:**
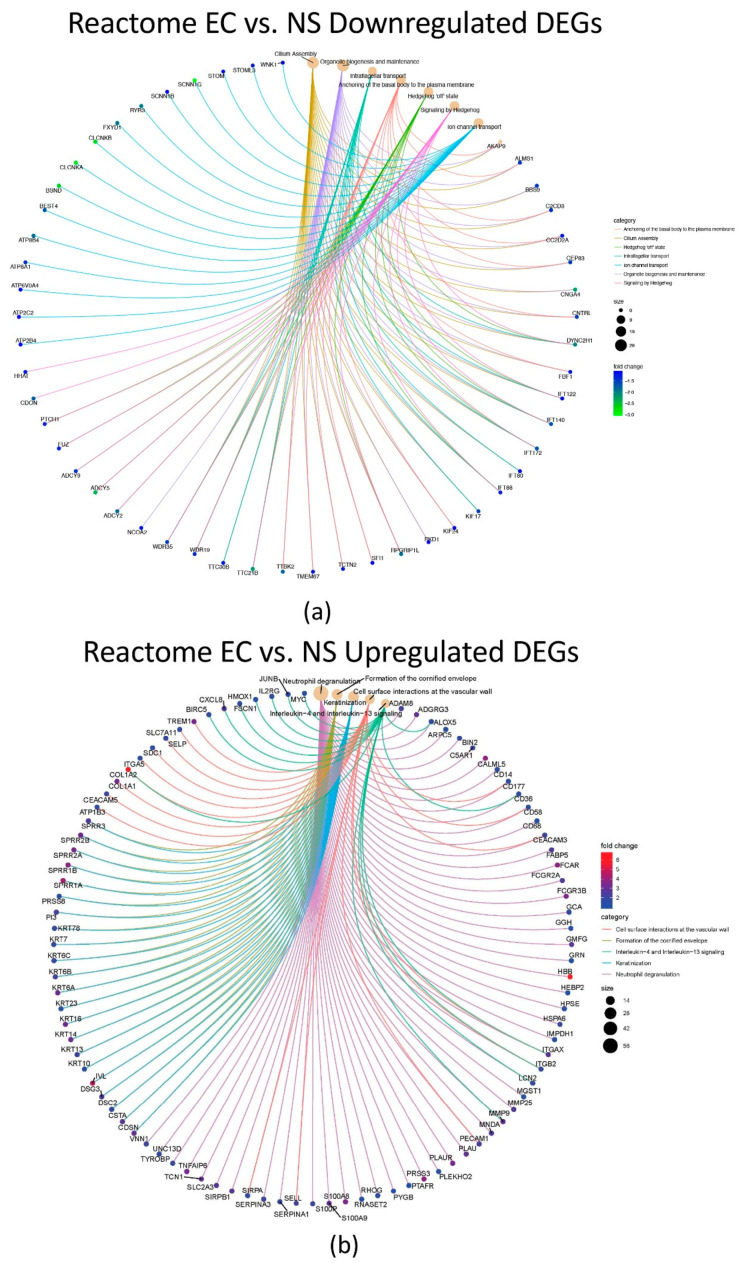
Top altered Reactome pathways and associated DEGs in the EC vs. NS set. (**a**) Pathways (light brown circles) enriched by downregulated DEGs in the EC vs. NS set. (**b**) Pathways (light brown circles) enriched by upregulated DEGs in the EC vs. NS set. Diagram shows the DEGs associated with each pathway and their fold changes (color key on right). Circle size key indicates the number of genes associated with each pathway. For (**a**), cilia assembly transcripts were down regulated. For (**b**), neutrophil degranulation was the major pathway. Images can be enlarged to read text.

Each comparison was also evaluated using Reactome enrichment analysis to identify the genes and pathways affected by smoking and EC use (Figure 3 and Figures S1–S3). In the EC vs. NS comparison, the downregulated DEGs enriched pathways included organelle biogenesis and maintenance, cilium assembly, signaling by hedgehog, anchoring of the basal body to the plasma membrane, intraflagellar transport, and hedgehog “off state” (Figure 3a and Figure S1a). The pathways identified with the upregulated DEGs of the EC vs. NS comparison included neutrophil degranulation, keratinization, formation of cornified envelope, cell surface interactions at the vascular wall, and interleukin-4 and 13 signaling (Figure 3b and Figure S1b). For CS vs. NS, the upregulated pathways included biological oxidations, metabolism of vitamins and cofactors, phase II—conjugation of compounds, and glucuronidation (Figures S1c and S2). Pathways for the downregulated DEGs were not identified in the CS vs. NS comparison. In the CS vs. EC comparison, four pathways were enriched by the downregulated DEGs, which included neutrophil degranulation, signaling by interleukins, keratinization, and formation of cornified envelope (Figures S1d and S3). No pathways were enriched by the upregulated DEGs.

### 3.5. Canonical Pathway Using IPA

There was one significant canonical pathway identified using a lung filtered data set for the EC vs. NS comparison, leukocyte extravasation signaling (z-score 2.668). For the CS vs. NS data, there were no pathways with significant z-scores; however, methylglyoxal degradation III, a relevant pathway, had significant *p*-value (<0.05). Only one canonical pathway with an activated z score was identified in the CS vs. EC comparison (LXR/RXR activation, z score 2).

### 3.6. Networks Identified in the EC vs. NS Comparison

**Figure 4:**
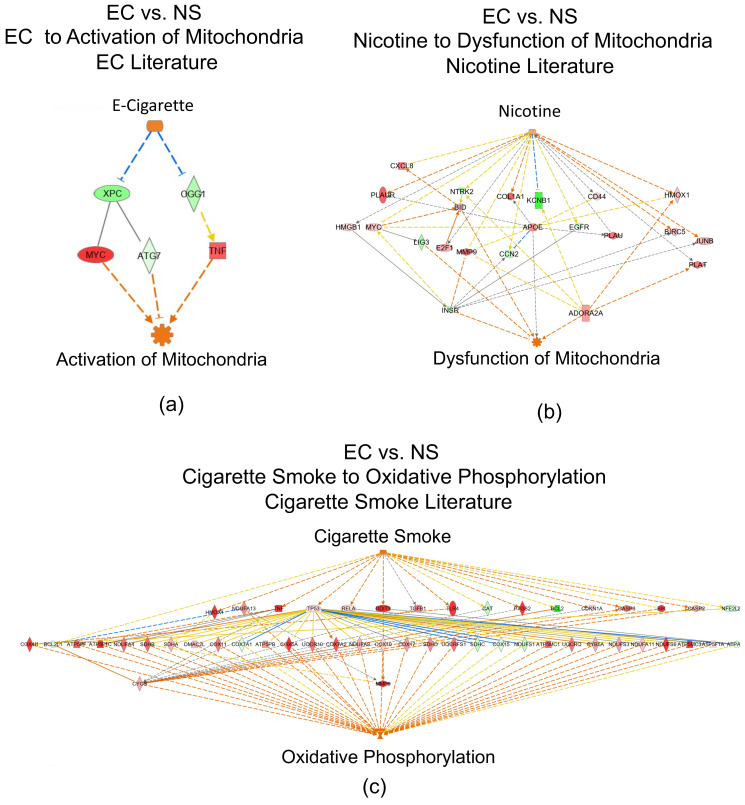
Mitochondrial pathways identified in the EC vs. NS set using IPA. (**a**) “Activation of Mitochondria” generated by overlaying our data on the EC literature. (**b**) “Dysfunction of Mitochondria” generated by overlaying our data on the nicotine literature. (**c**) “Oxidative Phosphorylation” generated by overlaying our data on the cigarette smoke literature. Upregulated DEGs are pink-to-red depending on fold-change, whereas green DEGs indicate downregulation. Orange arrows represent predicted activation, yellow arrows indicate non-consistent activation, and grey arrows represent effect not predicted. Images can be enlarged to read text.

**Figure 5:**
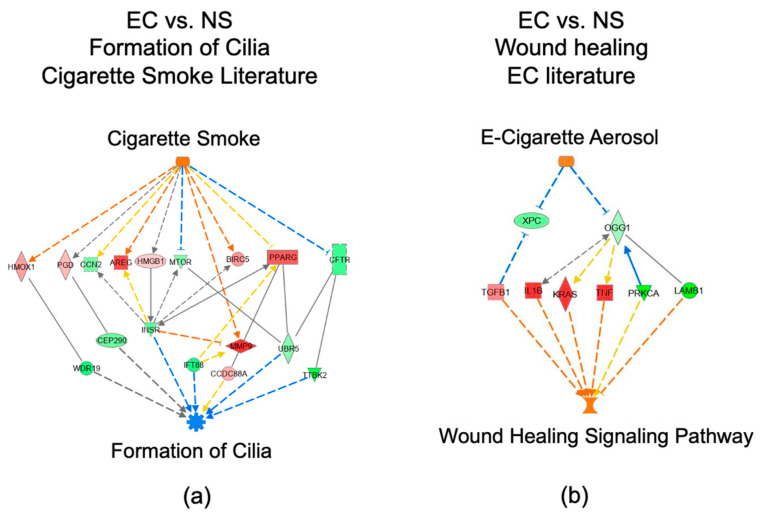
Pathways involved in formation of cilia and wound healing in our EC vs. NS set identified using IPA. (**a**) “Formation of Cilia” generated by overlaying our data on the cigarette smoke literature. (**b**) “Wound Healing Signaling Pathway” generated by overlaying our data on the EC literature. Upregulated DEGs are pink-to-red depending on fold-change, whereas green DEGs indicate downregulation. Orange arrows represent predicted activation, yellow arrows indicate non-consistent activation, and grey arrows represent effect not predicted.

DEGs in our data were compared to genes in the QIAGEN Knowledge Base to identify networks activated by “E-Cigarette Smoke”, “Nicotine”, or “Cigarette Smoke” using the “Path Explorer” tool in IPA (Figure 4). Pathway connections that were found included: (1) “E-cigarette Smoke” to “Activation of Mitochondria”, (2) “Nicotine” to “Dysfunction of Mitochondria”, and (3) “Cigarette Smoke” to activation of “Oxidative Phosphorylation” (Figure 4a–c). These networks are consistent with increases in mitochondria activity in the EC vs. NS group. Additional networks were also found linking “Cigarette Smoke” to inhibition of “Formation of Cilia” and linking “E-Cigarette Smoke” to activation of “Would Healing Signaling Pathway” (Figure 5a,b).

### 3.7. IPA Regulator Effect Analysis for the EC vs. NS Comparison

**Figure 6:**
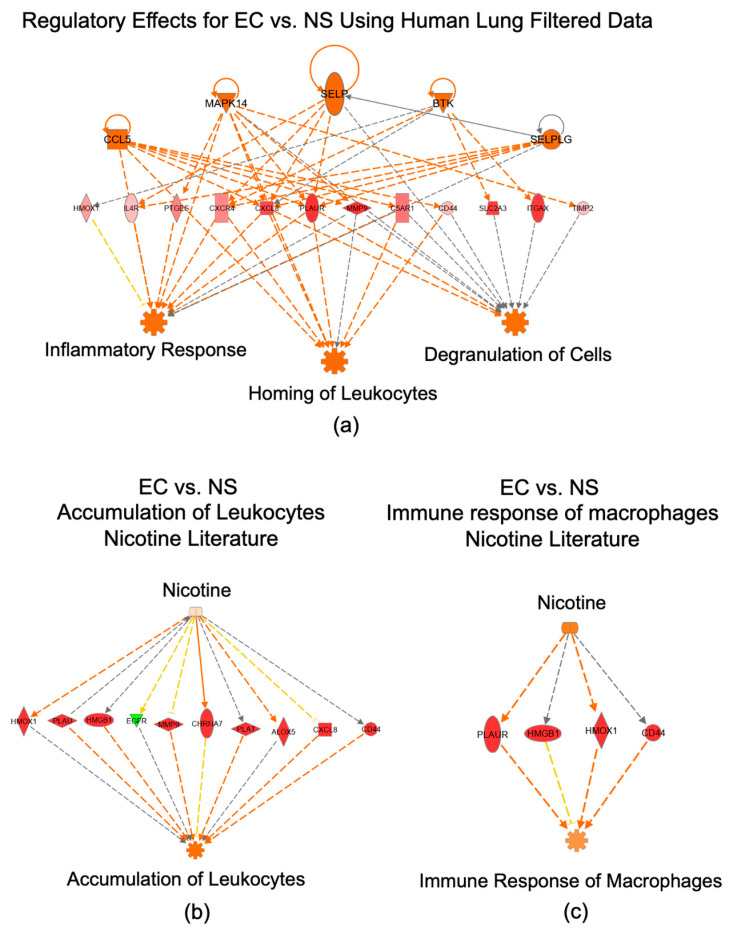
Evidence of an immune response in the EC vs. NS set. (**a**) IPA regulator effects analysis in the EC vs. NS set. Diagram shows predicted upstream regulators (upper row) and predicted downstream processes and functions (lower row) based on DEGs in our data (middle row). (**b**) “Accumulation of Leukocytes” generated by overlaying our data on the nicotine literature. (**c**) “Immune Response of Macrophages” generated by overlaying our data on the nicotine literature. Upregulated DEGs are pink-to-red depending on fold-change, whereas green DEGs indicate downregulation. Orange arrows represent predicted activation, yellow arrows indicate non-consistent activation and grey arrows represent effect not predicted.

The top regulator effect network, shown in Figure 6a, illustrates the five predicted upstream regulators, based on 12 DEGs in our data, and three major downstream effector functions related to inflammation that are predicted to be activated (inflammatory response, homing of leukocytes, and degranulation of cells) (Figure 6a). With IPA’s “Path Explorer” tool there was linkage between “Nicotine” and activation of “Accumulation of Leukocytes” and between “Nicotine” and activation of “Immune Response of Macrophages” (Figure 6b,c).

### 3.8. Disease Ontology Semantic and Enrichment (DOSE)

**Figure 7:**
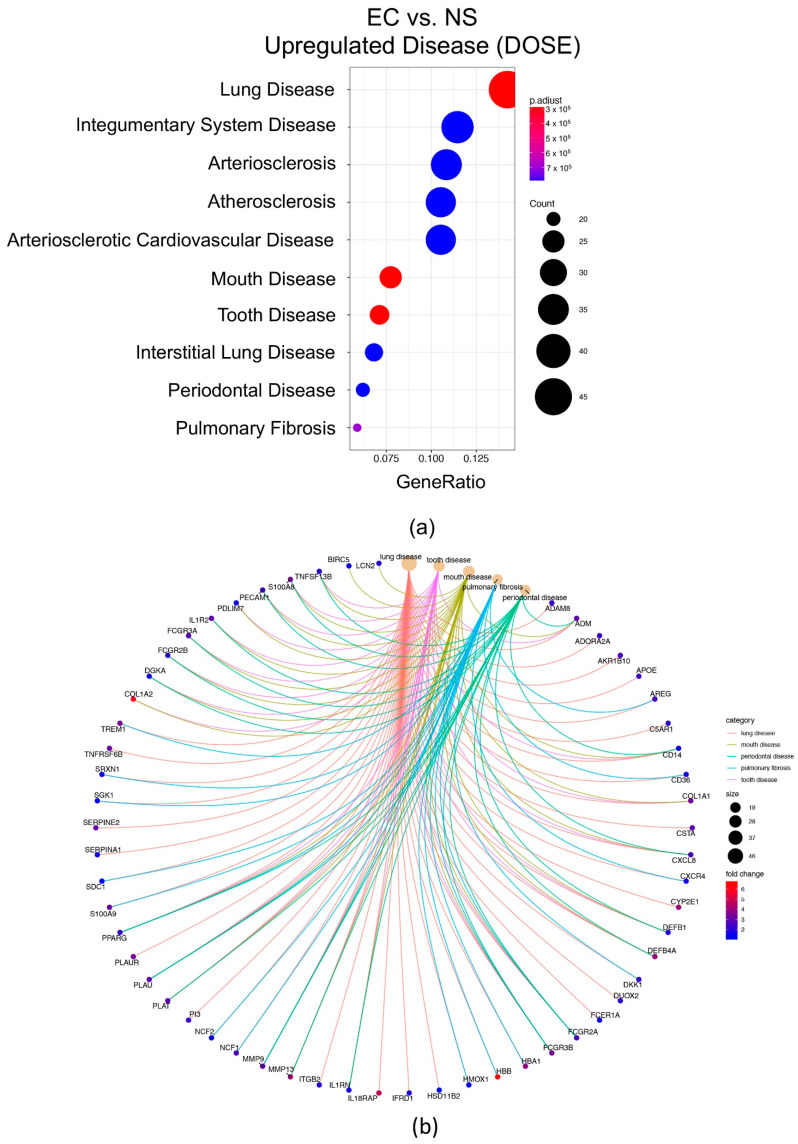
Disease Ontology Semantic and Enrichment (DOSE) analysis for upregulated genes in the EC vs. NS set. (**a**) Diseases plotted vs. gene ratio. Circles represent gene number associated with each disease and color represents the adjusted *p*-values. (**b**) Top enriched diseases in the EC vs. NS set include the respiratory system. Diagram shows the DEGs associated with each disease and their fold changes (color key on right). The circle size key indicates the number of genes associated with each disease. Images can be enlarged to read text.

DOSE analysis was performed for the DEGs that were down or upregulated in the EC vs. NS comparison. For the upregulated genes the disease categories related to the respiratory system included lung disease, mouth disease, tooth disease, periodontal disease, and pulmonary fibrosis (Figure 7a). The DEGs associated with these respiratory diseases were often overlapping and were upregulated in more than one disease (Figure 7b). For example, FCGR2A, FCGR3B, PPARG, PLAU and MMP9 were associated with five diseases (Figure 7b). The two major disease categories for the downregulated DEGs were primary ciliary dyskinesia (*p* adjusted) and developmental disorder of mental health (FDR).

## 4. Discussion

Nasal epithelium from former smokers who switched to ECs was characterized by increased oxidative stress, an inflammatory response, keratinization, and impaired cilia formation when compared to non-smokers. Similar alterations have also been associated with tobacco cigarette use [30,31,32,33]. In our study, the CS group had fewer transcriptomic alterations and was less robust than the EC group; nevertheless, cilia formation was decreased, and oxidative stress was increased in the CS group relative to the NS group.

Both the EC and CS groups showed evidence of oxidative stress (“NADPH oxidase” in EC and “Biological Oxidations” in CS). Oxidative stress via NADPH oxidase has been demonstrated in CS users and is associated with asthma, cystic fibrosis, chronic obstructive pulmonary diseases [34,35,36,37]. In our data, GO terms related to oxidative stress via NADPH oxidase were enriched in EC users, who showed increased expression of genes encoding cytoplasmic polypetide subunits (NCF1/p67phox, NCF2/47phox, NCF4/p40phox), which are necessary for the activation of the NADPH oxidase complex [38,39,40]. These cytoplasmic polypeptide subunits bind to the transmembrane region on NADPH oxidase, which activates the enzymatic complex to produce reactive oxygen species (ROS) [38]. The elevation of NADPH oxidase by EC use is supported by several other studies. NADPH oxidase genes (NOX1, NOX2, DUOX2) were upregulated in human bronchial epithelial cells exposed to cinnamon flavored ECs at the air-liquid interface [41]. Increased ROS levels associated with NOX2 elevation, a NADPH oxidase gene family member, was observed in the lungs of mice exposed acutely to EC aerosol [42]. The serum from non-smoking human subjects who vaped ECs increased ROS in human pulmonary microvascular endothelial cells by activating NADPH oxidase [43]. Lung injury in cigarette smokers has also been associated with increased ROS via NADPH oxidase [44].

Inflammatory signals induced by cigarette smoking were reverting or had reverted to non-smoker levels 14 days after cessation [45]. In our data, genes associated with inflammation (CXCL8, CXCL13 and IL 36G) were elevated 6 or more months after smokers had converted to EC use. This elevation of cytokines is likely caused by the EC aerosol per se since cigarette smoking cessation is associated with a decrease in these markers [45]. In addition, MMP9, an inflammatory regulator released by neutrophils and macrophages, was upregulated in our EC group, in agreement with increased MMP9 and elevated neutrophilic granule enzymes in sputum from EC users [46]. Degranulation of neutrophils is the major cause of tissue damage in smoking-related diseases, such as asthma and chronic obstructive pulmonary disease [47]. In our study, GO terms and Reactome pathways associated with activation and degranulation of neutrophils were enriched in the EC users. Inflammation caused by ECs has been linked to nicotine, glycerol, propylene glycol, and EC flavor chemicals [48,49,50,51]. Our data are consistent with the conclusion that ECs contain chemicals that maintain an inflammatory response, even after cigarette smoking has stopped.

Squamous metaplasia is a condition observed in the bronchial epithelia of smokers [52,53,54]. In smokers, squamous metaplasia may be partially reversible after smoking has stopped [55]. EC users in our study showed many characteristics of squamous metaplasia, including an increase in keratinization (keratin 10 and keratin 13), hyperproliferation of the epithelium, decreased expression of genes involved in ciliogenesis, and increase expression of involucrin [53,56]. Our data are consistent with the conclusion that squamous metaplasia existed in the EC group. EC use may interfere with recovery of squamous metaplasia in former smokers or may itself induce squamous metaplasia.

Accumulated evidence shows that both EC aerosol and cigarette smoke adversely affect cilia. In sheep, tracheal mucus velocity, an in vivo measure of mucociliary clearance, was reduced by EC vapor [57]. In a comparison of bronchial epithelium from former smokers vs. EC users who were former smokers, genes that are indirectly involved in ciliogenesis were downregulated in the EC group [19]. Our data extend these results by showing direct downregulation of genes involved in ciliogenesis and further compare the EC group directly to non-smokers. Our data support the conclusion that ciliogenesis had not returned to normal after switching from cigarettes to ECs. Several in vitro studies have examined EC chemicals that may impair cilia biogenesis. Human bronchial epithelial cells exposed to cinnamon-flavored EC aerosols had shorter cilia than their controls [41,58]. Flavor chemicals (diacetyl and 2,3-pentanedione) caused downregulation of genes involved in ciliary biogenesis (TEKT1, CFAP70, PROM1, DNAH12, DNAI1, DNAH3, DNAAF1, CC2D2A, CFAP221, SPAG17, and DNAH6) in mature primary human airway epithelial cells exposed in vitro [59]. The same set of genes was downregulated in our study suggesting the effect observed in our human subjects was caused by these flavor chemicals, which are frequently observed in EC products [60,61,62,63]. Cilia-related transcription factors (FOXJ1, RFX2, and RFX3) that were downregulated in our EC group were also downregulated in vitro in human airway epithelium exposed to cigarette smoke [33]. Our data are consistent with EC aerosols and cigarette smoke inhibiting ciliogenesis via similar target genes and further indicate that defective ciliogenesis was not reversed by switching from cigarettes to ECs.

There is an urgent need to identify diseases associated with EC use. Research directly connecting diseases to ECs is limited, and many studies in both animals and humans are based on acute exposures. Understanding the long-term human health effects of EC use could require decades, as was the case for cigarette smoke related diseases [64]. The human subjects in our study were chronically exposed to cigarette smoke (CS) or EC aerosol following smoking cessation (EC), and therefore give insight into both the phenotypes and diseases caused by these exposures. In the CS and EC group, the main phenotypes were dysregulation of cilia and increased oxidative stress. In the EC group, inflammation was also significantly increased. Ciliogenesis dysfunction can be indicative of ciliary dyskinesia syndrome, which inhibits clearance in the airways [65]. In the EC group, increased oxidative stress via activation of NADPH oxidase was a dominant phenotype and could lead to epithelial remodeling [66]. In the CS group, oxidative stress likewise was increased, as has been reported in other studies of human smokers. Elevated ROS in the lung leads to disease. such as respiratory distress syndrome, emphysema, and COPD, which are characterized by inflammation [67]. To further understand disease initiation and pathogenesis in our data, we took advantage of DOSE analysis. Many of the diseases enriched in the EC vs. NS comparison included respiratory-related diseases (lung diseases, interstitial lung disease, and pulmonary fibrosis), which are commonly associated with cigarette smoking. Here, we see that after switching from cigarette smoking to vaping for 6 months, evidence of lung disease was still apparent in the EC group. Other diseases detected in the DOSE analysis included the upper respiratory system (mouth disease, tooth disease, periodontal disease). These diseases have also been reported in cigarette smokers [68] and more recently in an epidemiological report on EC users [69]. Taken together our data support the conclusion that switching from cigarette smoking to EC use for 6 months does not restore the respiratory epithelium to that equivalent in the non-smoking group, suggesting that diseases that originate from oxidative stress, inflammation, and absence of cilia are present in the EC group.

Our study is affected by several factors. Our EC participants used second generation products that can operate at high powers, which may have generated aerosols that were more toxic than those produced by low powered fourth generation ECs. However, fourth generation products, such as JUUL^TM^, contain high concentrations of nicotine (~60 mg/mL) [70,71], flavor chemicals, and coolants [72] that could affect gene expression and/or protein levels, as shown in other tissues [51,73,74] and may produce results that differ from the current study. A greater number of DEGs were identified in the EC vs. NS set than in the CS vs. NS and CS vs. EC sets, similar to several previous studies [17,18]. The lower number of DEGs in CS makes this group less robust and may explain why fewer enriched terms and pathways were identified in the smokers. While exposures in the EC group were similar and represent what an average EC user would receive, the participants in the CS group were not exposed to equal numbers of cigarettes/day, which might have affected the identification of DEGs in the CS group. While we have found significant effects in both the EC and CS groups compared with the NS controls, future studies should be done using larger sample sizes that include males and a group of ex-smokers who do not use ECs.

## 5. Conclusions

Our data clearly show evidence of tissue damage in EC users who had stopped cigarette smoking for at least 6 months, indicating that switching from cigarettes to ECs does not restore the nasal epithelium to that of non-smokers. Both the EC and CS groups showed elevation of oxidative stress, evidence of ciliary dysfunction, and diminished ciliogenesis. Since cilia recovers in former smokers [75], the ciliary dysfunction seen in the EC group is likely due to chemicals in the EC aerosol. Formaldehyde and acrolein, reaction products produced during EC aerosol generation [76,77], are both irritants that inhibit ciliary beating [78] and may have compromised ciliary function in both the CS and EC groups. Compared to non-smoker controls, nasal epithelium from EC users also showed evidence of increased inflammation and squamous metaplasia. Since cytokines revert to non-smoker levels after 14 days of cessation [45] and squamous metaplasia also reverts following smoking cessation [54], it is likely that the elevation of inflammatory and squamous metaplasia markers that we observed was due to EC use per se. In the DOSE analysis, the DEGS in the EC group were linked to lung diseases, interstitial lung disease, and pulmonary fibrosis. EC aerosols are complex mixtures of chemicals that include nicotine [79,80], flavorants [70], metals [81,82], solvents, and reaction products [76,77], which are often present at high concentration and likely contributed to the observed effects. Our data are consistent with the conclusion that the chemicals in EC aerosols can produce effects that preclude restoration of the nasal epithelium to that of the non-smoking control group and that respiratory diseases may occur with prolonged use.

## Data Availability

FASTQ files can be accessed in the National Center for Biotechnology Information SRA database (PRJNA666452).

## Supplementary Materials

The following are available online at https://www.mdpi.com/article/10.3390/toxics10070370/s1, Figure S1: Reactome enriched pathways. (a) Enriched pathways upregulated in the EC vs. NS set. (b) Enriched pathways downregulated in the EC vs. NS set. (c) Enriched pathways upregulated in the CS vs. NS set. (d) Enriched pathways downregulated in the CS vs. EC set. (FDR < 0.05); Figure S2: Top altered Reactome pathways and associated DEGs in the CS vs. NS set. Pathways (light brown circles) enriched by the upregulated DEGs in the CS vs. NS set. Diagram shows the DEGs associated with each pathway and their fold changes (color key on right). Circle size key indicates the number of genes associated with each pathway; Figure S3: Top altered Reactome pathways and associated DEGs in the CS vs. EC set. Pathways (light brown circle) enriched by the upregulated DEGs in the CS vs. EC set. Diagram shows the DEGs associated with each pathway and their fold changes (color key on right). Circle size key indicates the number of genes associated with each pathway. Table S1. Demographics and Biomarker Data for Participants Included in the Transcriptomic Analysis; Table S2. E-Cigarette Users vs. Non-Smokers Differentially Expressed Genes; Table S3. Cigarette Smokers vs. Non-Smokers Differentially Expressed Genes; Table S4. Cigarette Smokers. vs. E-Cigarette Users_ Differentially Expressed Genes; Table S5. GO Terms for the EC vs. NS Comparison; Table S6. GO Terms for the CS vs. NS Comparison; Table S7. GO Terms for the CS vs. EC Comparison; Table S8. IPA Top Biological Functions for EC vs. NS using human lung filtered data.
