# Supplementary material for: Transcriptomic Evidence That Switching from Tobacco to Electronic Cigarettes Does Not Reverse Damage to the Respiratory Epithelium
Source: Toxics. 2022 Jul 4;10(7):370. doi: 10.3390/toxics10070370 (PMC9321508; doi:10.3390/toxics10070370)
Supplement: Supplementary file 1 [file toxics-10-00370-s001.zip › toxics-1712442-Supplementary-Figures.pdf]

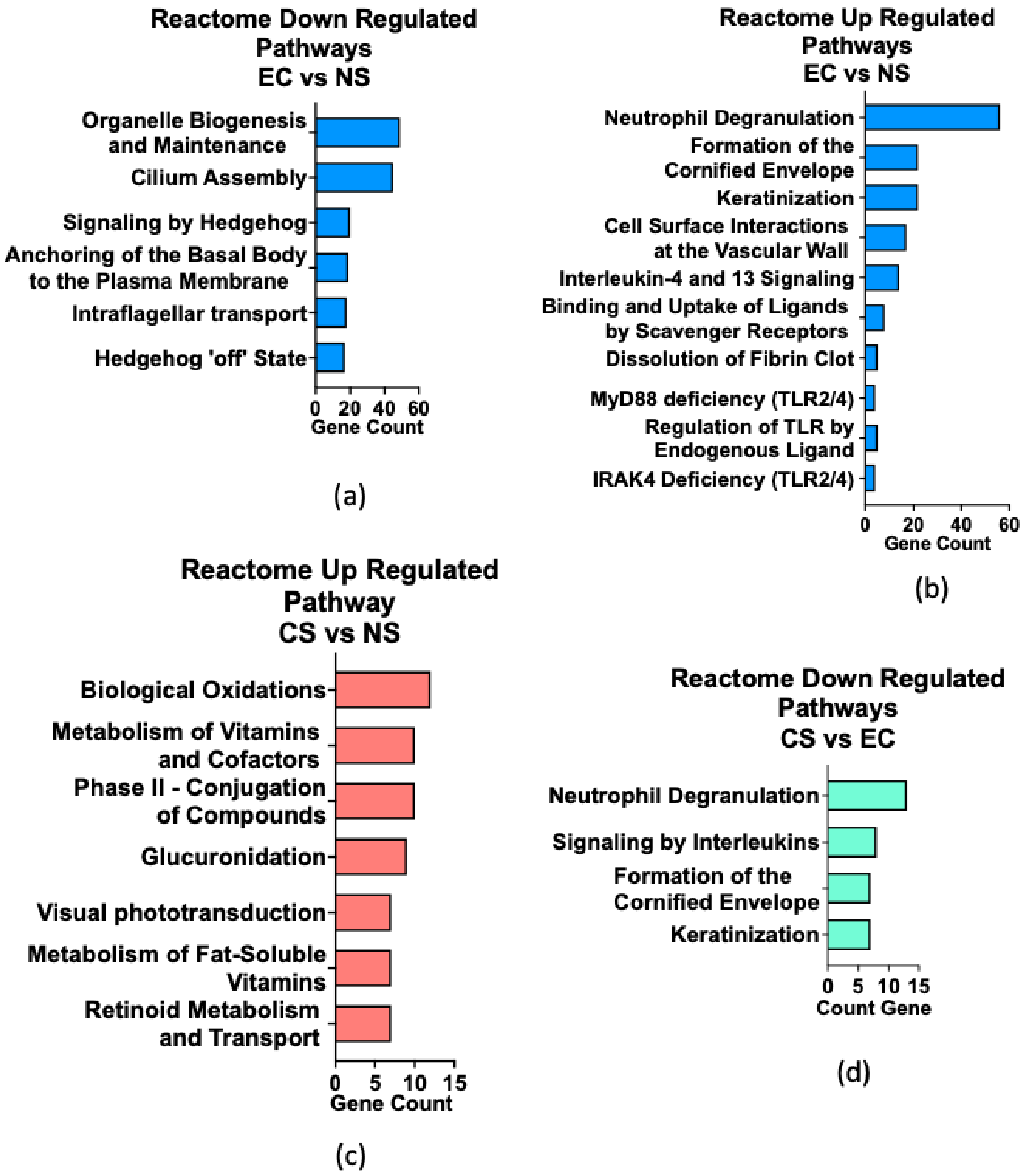

Figure S1. Reactome enriched pathways. (**a**) Enriched pathways upregulated in the EC vs. NS set. (**b**) Enriched pathways downregulated in the EC vs. NS set. (**c**) Enriched pathways upregulated in the CS vs. NS set. (**d**) Enriched pathways downregulated in the CS vs. EC set. (FDR < 0.05).

Reactome CS vs NS Upregulated DEGs

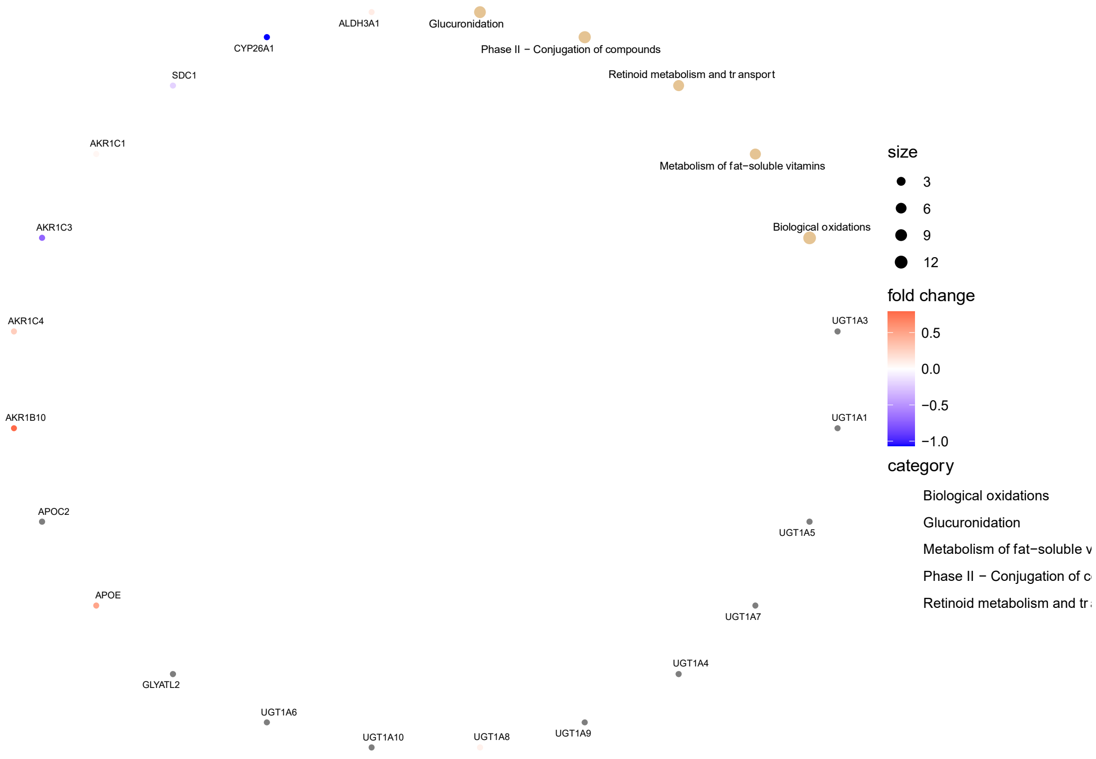

Figure S2. Top altered Reactome pathways and associated DEGs in the CS vs. NS set. Pathways (light brown circles) enriched by the upregulated DEGs in the CS vs. NS set. Diagram shows the DEGs associated with each pathway and their fold changes (color key on right). Circle size key indicates the number of genes associated with each pathway.

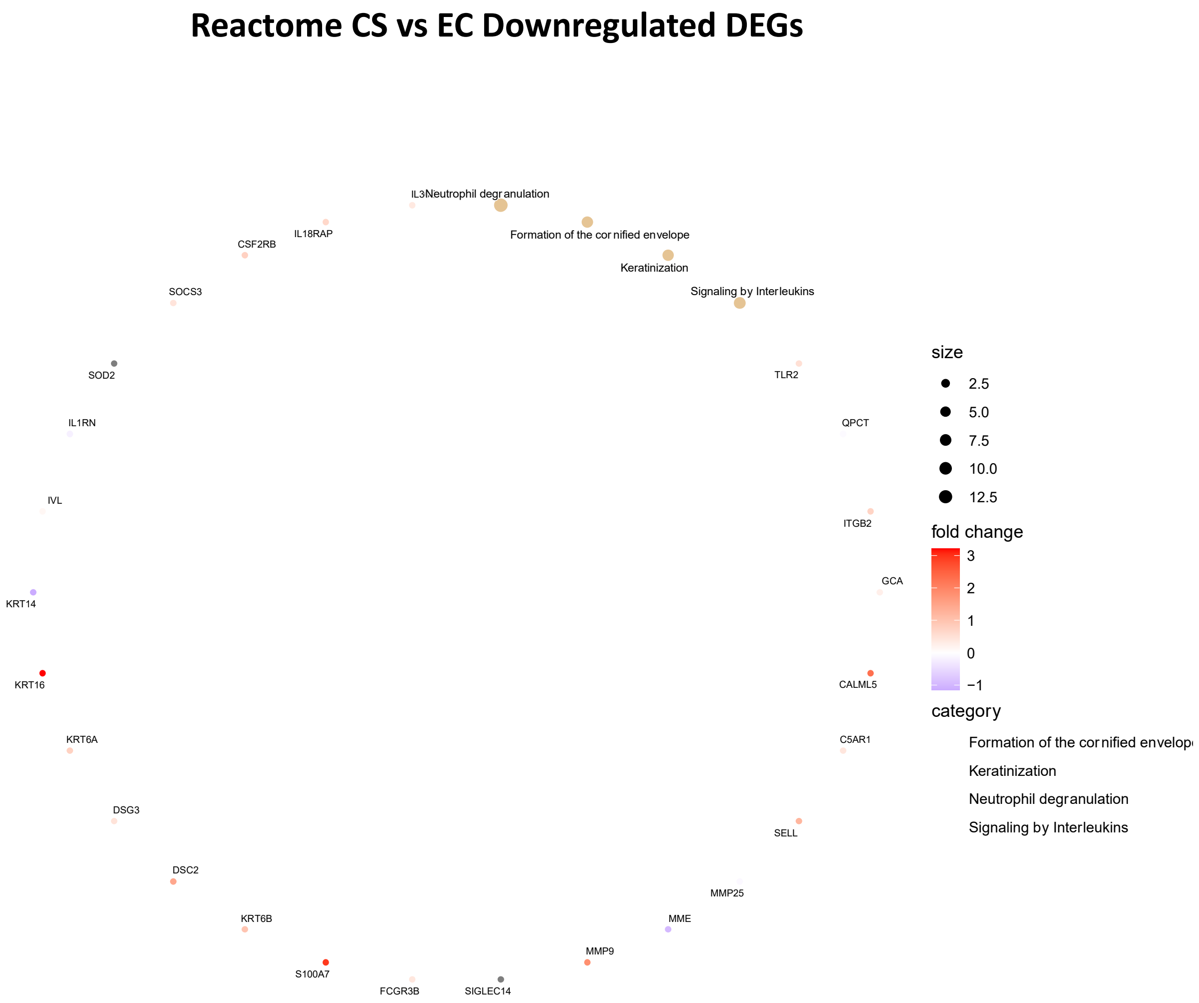

Figure S3. Top altered Reactome pathways and associated DEGs in the CS vs. EC set. Pathways (light brown circle) enriched by the upregulated DEGs in the CS vs. EC set. Diagram shows the DEGs associated with each pathway and their fold changes (color key on right). Circle size key indicates the number of genes associated with each pathway.
